# Uniform and Complete Irreversible Electroporation on Gastric Mucosa Enabled by Sintered Silver Hydrogel

**DOI:** 10.34133/cbsystems.0697

**Published:** 2026-10-05

**Authors:** Zhuoqun Li, Shenshen Lei, Quanpeng Li, Fulai Lin, Jian Lv, Yonghui Gong, Zhuomin Ping, Chengjiang Wang, Yuchen Cheng, Wenna Liu, Dingming Zhong, Yutang Li, Yuhao Wang, Feng Ma, Aihua Shi, Xiaoliang Chen, Chuncai Kong, Bai Sun, Sihai Luo, Jie Tao, Rongqian Wu, Hao Sun, Zheng Wu, Shuixiang He, Bowen Ji, Xinge Yu, Jinyou Shao, Fenggang Ren, Yi Lyu

**Affiliations:** ^1^ Department of Hepatobiliary Surgery, The First Affiliated Hospital of Xi’an Jiaotong University, Xi’an, Shaanxi 710061, China.; ^2^ Frontier Institute of Science and Technology (FIST), Interdisciplinary Research Center of Frontier Science and Technology, Xi’an Jiaotong University, Xi’an, Shaanxi 710049, China.; ^3^ Department of Biomedical Engineering, City University of Hong Kong, Hong Kong 999077, China.; ^4^ National Local Joint Engineering Research Center for Precision Surgery & Regenerative Medicine, The First Affiliated Hospital of Xi’an Jiaotong University, Xi’an, Shaanxi 710061, China.; ^5^ National Key Laboratory of Unmanned Aerial Vehicle Technology, Integrated Research and Development Platform of Unmanned Aerial Vehicle Technology, Unmanned System Research Institute, Northwestern Polytechnical University, Xi’an, Shaanxi 710072, China.; ^6^ Micro and Nanotechnology Research Center, State Key Laboratory for Manufacturing Systems Engineering, Xi’an Jiaotong University, Xi’an, Shaanxi 710049, China.; ^7^ Department of Gastroenterology, The First Affiliated Hospital of Xi’an Jiaotong University, Xi’an, Shaanxi 710061, China.; ^8^ School of Future Technology, Xi’an Jiaotong University, Xi’an, Shaanxi 710049, China.; ^9^ State Industry-Education Integration Center for Medical Innovations, Xi’an Jiaotong University, Xi’an, Shaanxi 710061, China.; ^10^ State Key Laboratory of Electrical Insulation and Power Equipment, Xi’an Jiaotong University, Xi’an, Shaanxi 710049, China.; ^11^ Ministry of Education Key Laboratory for Non-Equilibrium Synthesis and Modulation of Condensed Matter, Shaanxi Province Key Laboratory of Advanced Functional Materials and Mesoscopic Physics, School of Physics, Xi’an Jiaotong University, Xi’an, Shaanxi 710049, China.

## Abstract

Irreversible electroporation is a promising nonthermal ablation for gastric mucosa, but nonconformal contact between the electrode and the rough surface of the gastric mucosa causes an inhomogeneous distribution of the high-voltage electric field. Here, we enhanced gap filling and substantially reduced distortion of the delivered high-voltage electric field by fabricating a soft, highly conductive sintered silver hydrogel as the bioelectronic tissue interface, thereby achieving uniform and complete irreversible electroporation. The sintering reaction was initiated by Cl^−^ and H^+^, which can work synergistically to partially eliminate the insulating surfactant on silver flakes, facilitating a sintering reaction through the dynamic redeposition of silver nanoparticles on silver flakes. Via a room-temperature sintering process, the conductivity of the hydrogel was promoted from below 1 to >10^4^ S/m at a low silver flake loading (4 vol%), and the mechanical property was still kept at a low Young’s modulus (91 kPa), even after swelling from digestive juices. In vitro experiments demonstrated that ablation uniformity improved by up to 86%, with the ablation area increasing by 30% to 52% and a maximum depth of 5.39 mm. Preclinical validation using porcine models demonstrated uniform, complete gastric mucosal ablation, reaching a depth of 2.50 mm, extending through the muscularis mucosae and leaving no residual cells. This sintered silver hydrogel-enhanced irreversible electroporation addresses key challenges and reinforces its potential as a reliable, advanced treatment for gastrointestinal lesions.

## Introduction

The gastric mucosa serves as a barrier against outside pathogens and debris. It tends to develop into mucosal diseases and even cancer, which have high morbidity and a high risk of progression [1,2]. Current therapeutic strategies for gastric mucosal lesions, such as laparoscopic surgery or endoscopic submucosal dissection (ESD), are highly demanding in terms of technical expertise and carry a substantial risk of complications [3–5]. The main challenge is to completely treat the lesion while preserving the structural and functional integrity of the stomach. Therefore, it is highly desirable to seek more advanced and reliable treatment techniques for gastric mucosal lesions. Irreversible electroporation (IRE), also known as pulsed field ablation, utilizes the high-voltage pulsed electric field (PEF) to enhance cell membrane permeability and disrupt cell structural integrity [6,7]. IRE has been widely applied in tissue ablation due to its nonthermal process and tissue-selective features, which cause minimal thermal injury to collateral structures and help to preserve the extracellular matrix scaffold [8]. Consequently, an increasing number of studies have attempted to apply IRE via the endoscopic route to the gastrointestinal tract and other endoluminal scenarios [9–11]. Compared to conventional endoscopic treatment techniques, IRE enables endoluminal tissue ablation with less procedure time and effort. It reduces the risks of bleeding and perforation while offering precise control of the electric field over both the volume and the depth of ablation. Ultimately, the ablated mucosa undergoes comprehensive structural and functional remodeling facilitated by tissue regeneration [7]. Planar electrodes capable of achieving enlarged contact surface area with the mucosa are more compatible with the gastrointestinal tract than needle-based electrodes during IRE [12]. However, one of the most critical challenges to the efficacy of IRE is the occurrence of inhomogeneous and incomplete tissue ablation.

This problem stems from several factors, one of which is the nonconformal contact between the electrode and the innate uneven gastric mucosa, due to mismatched mechanical softness and incompatible charge transfer mechanisms [8,13]. The Young’s modulus of a conventional metal electrode (10^6^ to 10^9^ kPa) is orders of magnitude higher than that of the mucosa (10 to 10^3^ kPa) [14,15]. Even with adequate conductivity and stability, the mismatch in Young’s modulus hinders seamless contact, leading to gaps or vacancies between the electrode and mucosa, which results in substantial discrepancies in the delivered electric field between the mucosal surfaces at both macro- and microscale [16–18]. Consequently, this markedly reduces ablation uniformity and results in ablation failure [19,20]. The importance of ablation uniformity is particularly prominent for mucosal lesions, which can have irregular shapes and be larger in size [21]. Sufficient ablation depth within the gastrointestinal wall ensures ablative efficacy, while consistent depth guarantees a distinct ablation boundary and eliminates residual undesirable cells. Both factors directly influence the adaptability to various types of lesions and long-term therapeutic outcomes. The primary work has enhanced ablation electrodes on other soft tissue interfaces through balloon-based electrodes [22,23], conductive polymer coatings [24], and injectable poly(3,4-ethylenedioxythiophene)-based hydrogel electrodes [25]. However, these methods are not suitable for mucosal ablation, as the mucosal surface is much rougher and more dynamic, composed of gastric pits, covered with continuously secreted digestive juices. The aforementioned methods still struggle to achieve fully conformal contact [26]. Thus, establishing an effective electric field delivery interface between the electrode and the mucosa presents a substantial challenge for the IRE [27,28].

Hydrogel has emerged as an ideal material for bioelectronic tissue interface, capable of conformally adapting to the rough mucosal surface due to its softness, low Young’s modulus, and high wettability [29,30]. However, there is a lack of studies regarding the use of a hydrogel interface for gastric mucosa IRE via the endoscopic route. The hydrogel interface requires sufficiently high and appropriate electrical conductivity to ensure effective delivery of the high-voltage electric field [25]. However, common hydrogels utilize ions as charge carriers, and their ionic conductivity is too limited to deliver high voltages effectively [29,31]. A deficiency in electrical conductivity can weaken the PEF delivered to the targeted tissue, thereby compromising the ablation efficiency [29]. Efforts to endow the electrical conductivity of hydrogels have focused on incorporating conductive fillers, such as silver (Ag), liquid metals, and conductive polymers [25,32,33]. Among available conductive fillers, Ag flakes with a high aspect ratio have garnered attention for their excellent electrical conductivity, biocompatibility, and effective percolation networks at relatively low filler content [34]. However, there is a trade-off between the conductivity and mechanical integrity of the Ag-flake-based hydrogel, as excessive loading of Ag flakes increases electrical conductivity but deteriorates the cross-linking of the hydrogel networks, leading to particle leakage [35]. Meanwhile, the continuously secreted digestive juice of the gastric mucosa can swell the hydrogel and increase spacing among conductive fillers, ultimately causing a substantial decline in electrical conductivity [36]. Therefore, the hydrogel, with high electrical conductivity, mechanical integrity, and durability against digestive-juice-induced swelling, holds great potential to serve as an ideal bioelectronic tissue interface for gastric mucosa IRE.

Here, we report the first example of using a fabricated highly electrical conductive hydrogel as the bioelectronic tissue interface to enhance the uniformity and efficacy of gastric mucosal IRE via an endoscopic approach. The highly conductive hydrogel was fabricated by solution sintering the incorporated Ag flakes in the sodium alginate (SA)–acrylamide double networks. The sintering reaction was initiated by the Cl^−^ and H^+^, which can work synergistically to partially eliminate the insulating surfactant on Ag flakes, facilitating a sintering reaction through the dynamic redeposition of Ag nanoparticles on Ag flakes. The room-temperature sintering process in Ag flake hydrogel effectively removes the insulating surfactant from the Ag flakes and accelerates the dissolution/redeposition of Ag^0^ to Ag^+^, thereby promoting the hydrogel’s electrical conductivity from below 1 to >10^4^ S/m at a low Ag flake loading (4 vol%), while maintaining a low Young’s modulus (~91 kPa). The sintered Ag flake hydrogel interface, with high conductivity and flexibility, fills gaps between the electrode and mucosa, reducing the distortion of the delivered high-voltage electric field and contact impedance from 10^4^ Ω to nearly 10^2^ Ω by facilitating charge transfer between electrons (electrode) and ions (mucosa). More importantly, the uniformity of IRE increased up to 86%, the ablation area increased by over 30%, and the maximum ablation depth reached 5.39 mm compared to conventional methods in in vitro models. Finally, preclinical validation for mucosal IRE with the sintered Ag hydrogel interface in a porcine model showed that the interface enabled uniform, larger, and complete ablation zones (~2.50 mm in depth, covering muscularis mucosae) without residual viable cells. These findings suggest a substantial enhancement in the uniformity and efficacy of mucosal IRE, reinforcing its potential as a reliable and advanced treatment option for gastrointestinal mucosal lesions.

## Materials and Methods

### Simulation analysis

The simulation analyses were performed using the finite element analysis software COMSOL Multiphysics (version 6.2, COMSOL Co. Ltd., Burlington, MA, USA), following the previously published work [37]. The tissue models with grooves were isotropic. The surface of the tissue was carved with 1-mm-deep grooves occupied by simulated gas–liquid contents to mimic the rugae and the actual environment of the gastric mucosa (Figs. S1 and S2). The pulse voltage was set within a range of 100 to 600 V through a parametric study. In the simulation, the probability of tissue cell death was predicted using the Peleg–Fermi model, which correlates cell death with electrical injury induced by the electric field intensity and pulse number [38]. When the probability of cell death caused by electrical injury in a specific region is greater than 0.95, it can be treated as an effective ablation region [37]. The electrical conductivity of the tissue was set as 0.333 S/m [39]. All parameters related to the simulation are listed in Table S1.

### Synthesis of the hydrogel and the sintering solution

The Ag hydrogel was synthesized by the method described in a previous work with modifications [34]. A precursor solution was prepared by overnight blending acrylamide (A8887, Sigma-Aldrich, USA) and SA (180947, Sigma-Aldrich, USA) in deionized (DI) water at a 4:1 mass ratio. Then, Ag flakes (5 μm; BROFOS NANO, China) at a volume ratio of 4% and 20 μL of 0.3% calcium sulfate (EB000466, Energy Chemical, China) were mixed into 1 g of the precursor solution and stirred overnight. Subsequently, 16.67 μL of 2 wt% *N*,*N*′-methylenebisacrylamide (chemical cross-linker) (146072, Sigma-Aldrich, USA), 5.76 μL of *N*,*N*,*N*′,*N*′-tetramethylethylenediamine (accelerator) (T9281, Sigma-Aldrich, USA), and 10 μl of 5 wt% ammonium persulfate (initiator) (A3678, Sigma-Aldrich, USA) were added. The mixture was vigorously stirred and degassed to remove bubbles using a planetary centrifugal mixer (TM-310, Smida, China) and thermally reacted at 60 °C for 10 min to form the hydrogel network. The neat hydrogel was synthesized by the same method, except that Ag flakes were not added. The preparation of the sintering solution was adopted from the previous work [40,41]. The sintering solution was compounded with 100 mM NaCl and 17 mM lactic acid. After partial dehydration, the Ag hydrogel was soaked in sintering solution for 10 min and set aside in predesigned models overnight.

### Hydrogel characterization

Hydrogel morphology was observed by scanning electron microscope (SEM) (MAIA3 LMH, Tescan, Czech Republic). Hydrogel samples were frozen and dried overnight at −80 °C in a freezing dryer (LGJ-25D, Beijing Sihuan Qihang Technology Co. Ltd., China). Mechanical tests were performed on a universal mechanical test system (CMT-1103, Sansi Taijie Electrical Equipment Co. Ltd., China). Samples were prepared in a dog-bone shape and tested at a strain rate of 20 mm/min. Electrical properties were measured with a multimeter (Fig. S3). The hydrogel was cut into a rectangular shape of 60 mm in length, 10 mm in width, and 1 mm in thickness. Eutectic gallium indium (495425, Sigma-Aldrich, USA) was placed at the 2 ends to minimize the contact impedance between the hydrogel and the Ag tape. The multimeter (DAQ6510, Keithley, USA) cables were clamped to Ag tapes to measure the resistance of hydrogel samples. Body fluid stability was tested by soaking samples in artificial bodily fluids and measuring the changes in resistance. The hydrogel samples were cut into regular strips (60 mm in length, 10 mm in width, and 1 mm in thickness) and then soaked in simulated gastric fluid (SGF) (CW006, Chuangwei, China) and artificial intestinal fluid (AIF) (CW005, Chuangwei, China) for 10 h while recording resistance variation. The Ag flake leakage test was performed on a porcine stomach sample and skin sample, which were cut into squares of 30 mm × 30 mm. Hydrogels with different Ag contents were made into circles with a diameter of 25 mm. The hydrogel sheets were attached to the tissue sample with the same pressure and removed after 10 min to observe the Ag flake residue. Shear strength was measured on the basis of the standard lap-shear test (ASTM F2255) using the universal testing machine. The tensile speed was 5 mm/min. Fresh porcine stomachs were used as an ex vivo tissue model to evaluate the electrical stability of the hydrogel after ablation. The hydrogel samples were placed on the gastric mucosal tissue and subjected to the same ablation protocol as used in the in vivo experiments. After ablation, the hydrogel samples were collected, and their resistance was measured with a multimeter.

### Fabrication of the endoscopic ablation catheter system and the integration of the hydrogel interface

The IRE system included the magnetic-anchoring-assisted endoscopic ablation catheter system (EACS), an external magnetic anchoring device, an endoscope (EG-550, SonoScape, China), and an IRE system (A01, SurgeonsGate, China). The magnetic-anchoring-assisted EACS consists of 3 components: tip-mounted metal electrodes, a flexible catheter, and a cable operation unit. The metal electrode comprised a surface ablation circuit, a flexible thermoplastic polyurethane (TPU)-supporting substrate, and an embedded NdFeB magnet. The circuit was designed in the form of an oval ring. The TPU-supporting substrate for the metal electrode was designed with a ring on the backside to adjust the position under the endoscope by grasping forceps (Movie S2). The central electrode has a length of 11 mm, a line width of 2 mm, and a terminal fillet radius of 1 mm. The peripheral ring electrode has a major axis of 20 mm, a minor axis of 10 mm, and a line width of 2 mm. The major axis corresponds to the size of the mucosal lesion for which therapeutic endoscopic resection is an absolute indication (≤20 mm) [3,42]. The minor axis matches the diameter of the endoscope. The spacing between the ring electrode and the central electrode is also 2 mm. The electrode was made of double-sided copper flexible printed circuits with gold coating. The magnetic anchoring technique was accomplished by embedding an NdFeB magnet (20 mm × 10 mm × 2 mm) into the TPU-supporting substrate as the internal component magnet, which can be anchored to the surface of the gastric mucosa by an external magnetic device positioned from the abdominal wall.

The Ag/neat hydrogels were cross-linked in patterned silicone models cut by a cutting machine (Maker 3, Cricut, South Jordan) to control the shape and the thickness. The shape of the hydrogel was set as the same as the electrode, allowing it to completely cover the surface of the electrode. The hydrogel interface thickness for in vitro experiments is 1 or 2 mm, consistent with the grooves on potatoes. The hydrogel interface thickness for in vivo experiments is 0.6 mm, matching the gastric pits on the mucosa.

The hydrogels were partially dehydrated after synthesis and then swollen using a sintering solution. The metal electrode was plasma cleaned (CPC-Eplus, CIF, China) for 3 min to improve adhesion and integration with the hydrogel (Fig. S4). The flexible catheter consisted of 2 parts: an inner polytetrafluoroethylene catheter and an outer high-density polyethylene sheath. There is a length difference between the outer sheath and the inner catheter, thus endowing the flexible catheter with a certain degree of adjustability in its working length from 1,400 to 1,600 mm. The cable operation unit was fabricated by 3-dimensional (3D) printing to connect the generator with an adapter cable.

### Tissue filling and electrochemical measurements

The porcine stomach samples harvested from the abattoir were cut into 20 mm × 20 mm squares. Metal electrodes with a sintered Ag hydrogel interface and bare metal electrodes were attached to the mucosa and clamped with 2 slides to simulate tissue contact under in vivo conditions. The electrode-tissue interface was photographed with an optical microscope (AO-AF830R, AOSVI, China). Electrochemical impedance spectroscopy involves applying an alternating current voltage across the electrodes and measuring the resulting current response. These electrodes were positioned on porcine gastric mucosa and connected to an electrochemical workstation (Squidstat Plus, Admiral, USA) for precise measurements. Impedance measurements were performed across a frequency range of 1 to 10^6^ Hz to capture a comprehensive profile of the bioelectrical properties.

### In vitro potato ablation

Potato samples were cut into 20 mm × 30 mm rectangular pieces with an average thickness of 10 mm for the in vitro experiment. The surfaces in contact with the electrode were scribed with varied grooves using a 3D-printed mold to simulate the morphology of the mucosal surface. The grooves on the potato model had 2 varied parameters, including the depth (denoted as *D*) and the width (denoted as *W*), to simulate different morphologies of the gastric mucosa. Three experimental groups were established: metal electrode with a sintered Ag hydrogel interface, metal electrode with a neat hydrogel interface, and bare metal electrode. All kinds of electrodes were applied to the center of the carved simulation surface with an identical and constant pressure during the ablation. The pulsed voltage was set at 100, 150, 200, 250, and 300 V and maintained for 100 s, including 100 pulses with a duration of 10 μs. Before and after ablation, impedance measurements were repeated on the samples to assess changes in bioelectrical properties. After the ablation, the potato cubes were left at 4 °C for 24 h to promote melanin accumulation. Then, potato cubes were cut along the central axis of the ablation zone. The cross-sectional images were photographed under consistent lighting and angles using a camera (EOS D90, Canon, Japan) and analyzed in ImageJ software (ImageJ 1.54 g, National Institutes of Health, USA). The ablation depth ratio, ablation area, and the maximum ablation depth were measured for all the samples.

### Preclinical porcine experiments

All animal experiments were conducted under the approval of experimental protocols (XJTU2022-524) by the Committee on Animal Care at Xi’an Jiaotong University. The study included 3 experimental groups: the metal electrode with sintered Ag hydrogel interface, the metal electrode with neat hydrogel interface, and the bare metal electrode. Three male swine (Junxing, China) weighing 80 to 90 kg were used for in vivo mucosal IRE. The swine were placed on a liquid diet 24 h before the study and fasted on the day of the procedure. Before the procedure, the swine were sedated using intramuscular injection of 7 mL of tiletamine and zolazepam (Zoletil 50, Virbac, France) and intravenous injection of 50 mg of propofol (Peifen, JIABO, China). After intubation, anesthesia was maintained with isoflurane inhalation (2% oxygen). Under anesthesia, vital signs were monitored and recorded throughout the procedure. The EACS was positioned at the gastric body under endoscopic guidance, maintaining uniform and constant contact force between the electrode and mucosa by magnetic anchoring. Each swine was ablated at 3 points on the anterior wall of the stomach. Electrodes with 3 different interface conditions were used for each swine to minimize the influence of individual swine characteristics on the results. The pulsed parameters were a width of 10 μs and a frequency of 1 Hz, with voltages set at 500 V. A total of 50 pulses were performed at each point. The magnetic anchoring technique ensures that the electrodes are stably fixed at the target position. Swine were euthanized with pentobarbital sodium 1 h after ablation for harvesting the ablated tissue by laparotomy for histopathological examination. 

### Statistical analyses

The Shapiro–Wilk test was used to assess the normality of the distribution of continuous variables. Normally distributed continuous variables were expressed as means ± SD, and comparisons were performed using the 2-way analysis of variance (ANOVA) (GraphPad Prism 10.1.2). A 2-sided *p* < 0.05 was deemed statistically significant throughout the study.

## Results

### Design of the sintered Ag hydrogel interface for uniform and complete mucosal IRE

Endoscopy is the most advanced and convenient method for the early diagnosis and treatment of gastrointestinal mucosal lesions (Fig. 1A-i). As the first-line endoscopic treatment technique, ESD enables en bloc resection of mucosal lesions while preserving the integrity of the stomach. However, it has long been plagued by prolonged procedure times, a high risk of complications, and a long learning curve (Fig. 1A-ii). IRE represents an emerging nonthermal modality with considerable potential for the targeted treatment of superficial gastric lesions, characterized by a reduced complication rate and a minimally invasive procedure (Fig. 1A-iii). However, the inherent roughness of the mucosal surface poses a challenge to achieving uniform and complete ablation because of the nonconformal contact between the electrodes and mucosa. Although increasing the voltage can expand the ablation area, excessively high voltage can cause undesirable adverse events. This problem may be addressed through approaches such as combination therapy and interface optimization [43].

**Fig. 1.**
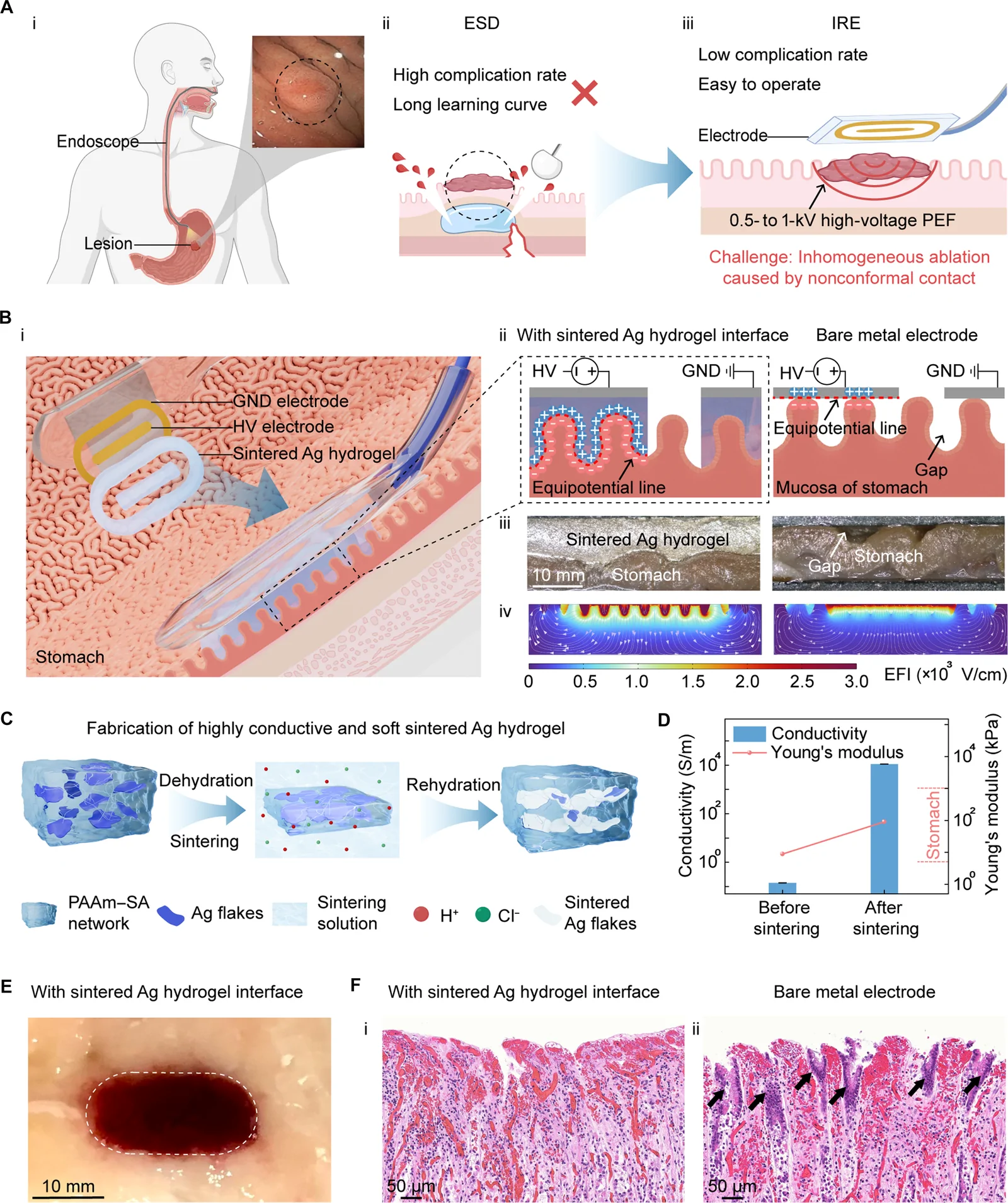
Design of the sintered Ag hydrogel interface for uniform and complete mucosal irreversible electroporation (IRE). (A) (i) Schematic of endoscopic examination for the gastric mucosa and the appearance of a gastric mucosal lesion. (ii) Schematic of the procedure of endoscopic submucosal dissection (ESD). (iii) Schematic of mucosal IRE (created in BioRender). (B) (i) The designed concept of mucosal IRE with a sintered Ag hydrogel interface on the mucosa. (ii) The distribution of equipotential lines under different interface conditions (GND, ground; HV, high voltage), (iii) the conformal effect of soft hydrogel between the electrode and mucosa, and (iv) the distribution of electric field intensity (EFI) on the mucosa under different interface conditions by simulation. (C) Schematic of room-temperature sintering process, including dehydration and rehydration in sintering solution processes (created in BioRender). (D) Conductivity (S/m) and Young’s modulus (kPa) of the sintered Ag hydrogel before and after sintering. (E) The top-view optical images of the mucosal surface ablated by the metal electrode with a sintered Ag hydrogel interface (a white dashed line circles the ablation boundary). (F) Hematoxylin and eosin (H&E) staining of ablated mucosa under different interface conditions [(i) with a sintered Ag hydrogel interface and (ii) without an interface].

Here, we propose the design of the gastric mucosa IRE with a sintered Ag flake hydrogel serving as the interface between the metal electrode and the mucosa (Fig. 1B-i). To improve the controllability of electrode manipulation within the stomach and establish a stable electrode-mucosa interface, we used a magnetic-anchoring-assisted technique to assist device manipulation. As shown in Fig. S5, an internal magnetic component was built into the electrode and works with an external magnet to fix the ablation electrode. At the electrode-mucosa interface, a highly conductive hydrogel can eliminate gas or liquid gaps, enabling consistent and continuous distribution of equipotential lines on the mucosal surface (Fig. 1B-ii). The local magnification imaging shows that the softness of the hydrogel interface enables conformal contact with rough mucosa (Fig. 1B-iii). Compared to the bare metal electrode, the sintered Ag hydrogel eliminated dead space between the electrode and mucosa, enabling conformal contact with the rough topography of the mucosal surface. The high electrical conductivity ensures effective electric field delivery without attenuation (Fig. 1B-iv).

In general, it is challenging to endow hydrogel with high electrical conductivity because the volume ratio of conductive filler is too low to form a dense conductive network. Increasing the volume ratio of conductive filler alone to enhance electrical conductivity would impede cross-linking of the hydrogel and compromise the mechanical integrity. Moreover, the hydrogel swelling in the digestive juice would increase the distance among conductive fillers, reduce the density of the conductive network, and deteriorate electrical conductivity. As shown in Fig. 1C, for the first time, we designed the room-temperature sintering reaction among Ag flake conductive fillers to endow the hydrogel with high electrical conductivity, mechanical integrity, and durability in a body fluid environment. Ag flakes with excellent electrical conductivity and a high aspect ratio serve as the filler, providing electron transfer pathways, while the sintering reaction weakens the barrier for the fluent electron transfer [34]. Sintering reaction is an effective way to enhance the electrical conductivity in Ag-based conductors by removing the insulating surfactant and bridging the adjacent Ag conductive fillers. However, thermal heating processes used in common sintering treatments restrain their deployment in hydrogel-based Ag conductors [44]. In our work, room-temperature sintering of Ag flake hydrogel was performed by soaking the partially dried hydrogel in an aqueous solution containing Cl^−^ and H^+^. The Cl^−^ and H^+^ work synergistically to partially remove the insulating fatty acid surfactant and enhance the electron transfer among Ag flakes, increasing the conductivity of the hydrogel from <1 to >10^4^ S/m (Fig. 1D). The Young’s modulus of sintered Ag flake hydrogel (~91 kPa) is within the range of mucosa (10 to 10^3^ kPa), enabling the complete filling in gastric pits with the presence of pressure, as shown in Fig. S6. The fully conformal contact between the highly conductive sintered Ag hydrogel and the gastric mucosa contributes to the effective delivery of the generated PEF to the targeted area.

The efficacy of introducing the sintered Ag hydrogel has been validated in preclinical studies of gastric mucosal IRE via endoscopy in a porcine model. The boundary of ablation zone on the mucosal surface (presents as dark red) was regular, clear, and distinct from adjacent normal tissue (Fig. 1E). Meanwhile, the electrode with a sintered Ag hydrogel interface could achieve complete ablation even at the gastric pit areas (Fig. 1F-i), whereas the bare electrode with conventional interface only achieved incomplete ablation with residual intact cells in intrusive areas (Fig. 1F-ii). These results demonstrated that the uniformity and efficacy of mucosal IRE via endoscopy were markedly enhanced with the contribution of sintered Ag hydrogel as the electrode-mucosa interface.

### Simulation analysis of conductive conformal interfaces for IRE

The finite element analysis was used to investigate the effect of a conformal interface between the electrode and the rough gastric mucosa on the electric field distribution. Meanwhile, the correlation between the electrical conductivity of the interface material and ablation volume was also studied. The simulation model consisted of a tissue cube, a pair of metal electrodes, and an electrode-mucosa interface (Fig. 2A and Figs. S1 and S2). The metal electrodes were patterned in a concentric elliptical configuration, in which the central electrode was set to have a high-voltage potential, and the outer electrode was grounded. The electrode-mucosa interface includes 3 different conditions: the conductive conformal interface, the gas–liquid interface, and the bare metal electrode condition.

**Fig. 2.**
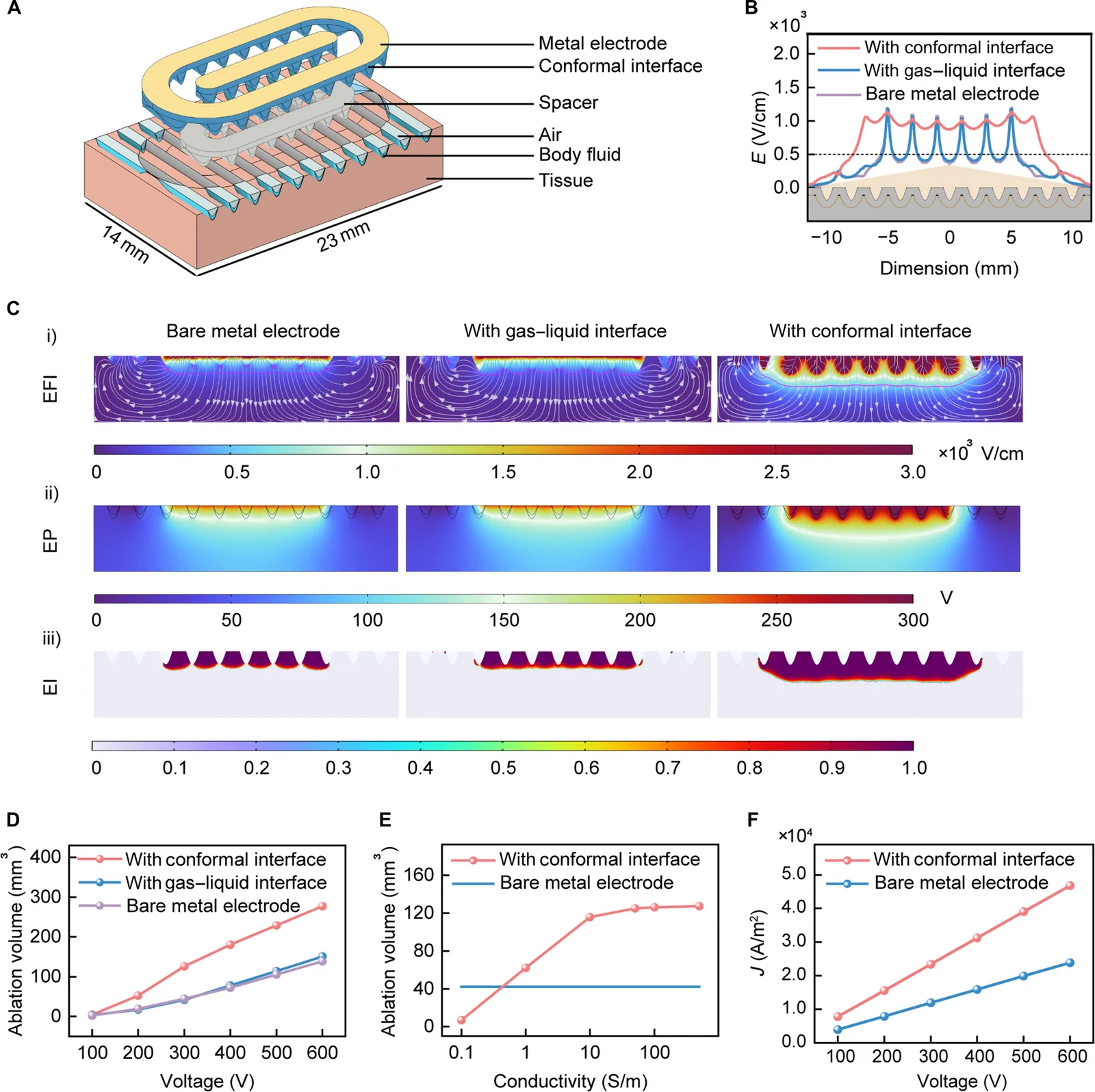
Simulation analysis of the properties of conductive conformal hydrogel interface. (A) The structure of the simulation model. (B) The electric field intensity (EFI) of a line 0.6 mm from the surface in the central area with the presence of a 300-V mounted voltage. (C) (i) EFI, (ii) electric potential (EP), and (iii) electrical injury (EI) in the cross-section of the model. (D) Ablation volumes of tissue ablated by the metal electrodes under different interface conditions. (E) The ablation volume of metal electrodes with conductive interfaces with varied electrical conductivities (from 10^−1^ to 10^2^ S/m). (F) The current density on the tissue surface under different pulse voltages.

The introduction of a conductive and conformal interface increased the uniformity of the electric field distribution and the effective ablation area (Fig. 2B). The electric field intensity at a depth of 0.6 mm from the tissue surface was selected to evaluate the electric field uniformity under different interface conditions. In this study, 500 V/cm has been set as the threshold electric field intensity for cell death caused by IRE. The conductive conformal interface can achieve the most uniform electric field distribution, including protrusive and intrusive areas. Furthermore, the area where the electric field intensity exceeds 500 V/cm is much larger. However, the electric field uniformity of the bare metal electrode is the worst, with electric field intensities around the trough regions falling below 500 V/cm. These areas, particularly in concave regions, are highly susceptible to localized distortions in the electric field distribution. Though the simulated gas–liquid interface can enhance the electric field intensity, the effect was minor because of its low ionic conductivity and partial filling. The soft conductive interface can simultaneously improve the uniformity and enlarge the ablated area through its conformal contact and effectiveness in delivering the electric field.

Fig. 2C shows the distribution of the electric field (Fig. 2C-i), electric potential (Fig. 2C-ii), and electrical injury (Fig. 2C-iii) at the central cross-section of the model under different interface conditions. The highly conductive conformal interface (*σ* = 100 S/m, 1 mm in thickness) can deliver the mounted 300-V electric field to the bottom of the grooves. Still, the intensive electric fields only appeared on the top of the grooves under the condition of a bare metal electrode (Fig. 2C-i). Fig. 2C-ii also shows that the electric potential distribution of the electrode with a conductive conformal interface was much larger. The gaps in grooves act as a barrier for the effective delivery of the electric field. Fig. 2C-iii shows the distribution of electrical injury. This indicates that the bare metal electrode primarily caused injury in the protrusive area, with discontinuous injury boundaries, whereas the conductive conformal interface resulted in uniform injury across the rough tissue, covering the largest area, with smooth and straight injury boundaries.

Moreover, the total ablation volume under the condition of a conductive conformal interface is markedly larger than that of others (Fig. 2D). The conformal contact and sufficient conductivity construct an outstanding interface that delivers a mounted electric field to rough tissues with minimal attenuation. Furthermore, we investigated the optimal electrical conductivity of the conformal interface (Fig. 2E). The ablation volume remains stable at 126 mm^3^ when the conductivity exceeds 10^2^ S/m, as the electron transfer pathway is adequate to deliver the mounted voltage. Therefore, for practical ablation, the electrical conductivity of the interface should be maintained above 10^2^ S/m. As shown in Fig. 2F, the current densities with the increase in pulse voltage are higher for the conductive conformal interface than for the bare metal electrode conditions, indicating reduced interface impedance and the transfer of increased effective charge at the same voltage.

In summary, the simulation analysis demonstrated that the conformal conductive interface with sufficient electrical conductivity can theoretically improve the electric field uniformity, the ablation volume, and the ablation depth of the IRE by maintaining conformal contact and effectively delivering the electric field.

### Synthesis and properties of the sintered Ag hydrogel

The soft and highly conductive conformal interface material was synthesized by immersing the Ag hydrogel into a sintering solution containing 100 mM NaCl and 17 mM lactic acid. The polyacrylamide (PAAm)–SA double-network hydrogel was chosen as the matrix due to its superior toughness and stretchability, which are facilitated by the mechanical and chemical interactions between the 2 matrices (Fig. S7) [45]. Ag flakes were utilized as conductive fillers because of their excellent biocompatibility, high conductivity, scalability in fabrication, and ease of forming conductive percolation networks [34]. However, incorporating Ag flakes into the hydrogel matrix alone is insufficient to achieve high electrical conductivity, as the volume ratio of conductive fillers is too low to establish adequate conductive networks, particularly when accounting for swelling in the wet stomach. The key point to endow enhanced electrical conductivity is the ambient sintering reaction initiated by the Cl^−^ and H^+^. These ions can work synergistically to partially eliminate the insulating surfactant on Ag flakes, facilitating a sintering reaction through the dynamic redeposition of Ag nanoparticles on Ag flakes [40,41].

The sintered Ag hydrogel with 4 vol% of Ag flakes was utilized as the conformal interface material due to its overall excellent mechanical integrity, sufficient conductivity, and electrical stability in body fluids. Fig. 3A shows that the sintered Ag hydrogel was patternable and could be elongated to nearly 100% strain without failure, suggesting its capability to be patterned into shapes that cover predesigned metal electrodes. The sintering process involved the partial dehydration of the Ag hydrogel and subsequent soaking in the sintering solution. Partial dehydration can facilitate intimate contact and connection between adjacent Ag flakes, allowing for the formation of electrically conductive pathways [34]. The electrical resistance of Ag hydrogels with varying Ag contents during partial dehydration demonstrated that water evaporation increased the electrical conductivity over time (Fig. S8). Thereafter, the rehydration in sintering solution induced changes in both the hydrogel networks and Ag flakes (Figs. 3B and C and Fig. S9). The hydrogel swelling in the sintering solution caused the formation of larger pores, and the capillary force generated by absorbed water can increase the distance between adjacent Ag flakes. This led to markedly increased resistance when the Ag hydrogel was soaked in DI water, from 0.7 Ω to 6.7 × 10^4^ Ω (Fig. 3D). Despite having a similar swelling ratio, the rehydration in sintering solution resulted in a minimal resistance increase, from 1 to 4.7 Ω after 1 h of swelling (Fig. S10). This phenomenon was probably due to the sintering reaction on Ag flakes. Cl^−^ and H^+^ can work synergistically to remove insulated surfactants and increase the connections of adjacent Ag flakes. Meanwhile, Cl^−^ has a high affinity for Ag^+^, which accelerates the dissolution/redeposition of Ag^0^ to Ag^+^, thereby enhancing the electrical conductivity of the Ag hydrogel [40,41,46]. As shown in high-magnification SEM images (Fig. 3C), the insulating surfactants were removed, and Ag flakes were exposed on the surface.

**Fig. 3.**
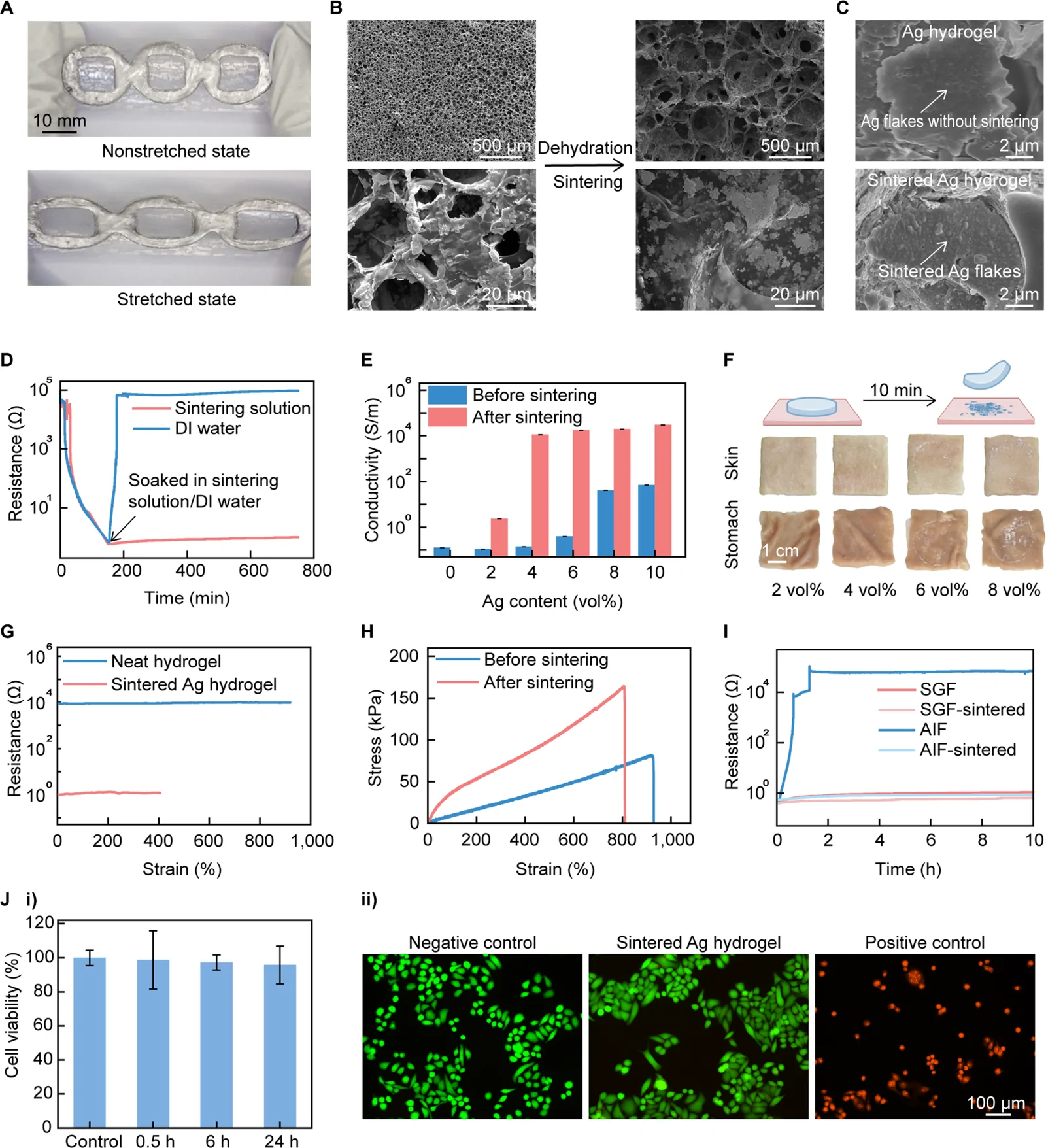
Synthesis and characterization of the sintered Ag hydrogel for the electrode-mucosa interface. (A) Optical photos of the sintered Ag hydrogel in the normal state and stretched state. (B) The scanning electron microscope (SEM) images of Ag hydrogel before (left) and after (right) sintering. (C) High-resolution SEM images of sintered Ag hydrogel (the arrows point to the surface of the Ag flakes). (D) The variations in resistance during dehydration and rehydration in the sintering solution. (E) Conductivity changes of Ag hydrogels with varied contents before and after sintering. (F) Ag leakage test on the porcine skin and stomach (the illustration was created in BioRender). (G) The resistance changes of the sintered Ag hydrogel and the neat hydrogel during extreme stretching. (H) Stress–strain curves of the Ag hydrogel before and after sintering. (I) Resistance variations of Ag hydrogel in simulated gastric fluid and artificial intestinal fluid before and after sintering. (J) (i) Cell viability of GES-1 cells in sintered Ag hydrogel extract at different coincubation times and (ii) live/dead viability fluorescence images of negative control, sintered Ag hydrogel, and positive control groups. Green fluorescence indicates viable cells, while red indicates dead cells.

Using the sintering solution to treat the dehydrated Ag hydrogel can achieve higher conductivity with a relatively lower Ag content, thus reducing the risk of Ag flake leakage. Before the sintering process, the conductivity of Ag hydrogel with increased Ag contents (0 to 10 vol%) was gradually elevated (Fig. 3E). The conductivity of Ag hydrogels with 2 and 4 vol% Ag contents was under 10^−1^ S/m, indicating the dominance of ionic conductivity rather than electronic conductivity. When the Ag content was increased to above 4 vol%, the Ag flakes formed a conductive network, resulting in an increase in electrical conductivity to 10^2^ S/m. However, when the Ag content exceeded 4 vol%, visible leakage of Ag flakes appeared when the hydrogel was pressed on the surface of porcine stomach and skin (Fig. 3F). The sintering process can boost the electrical conductivity from 10^−1^ S/m to >10^4^ S/m with Ag content at 4 vol%, indicating that electrically conductive pathways were formed. With the merits of the sintering process, Ag hydrogel can simultaneously realize the desirable electrical conductivity and prevent the Ag flake leakage caused by overloading.

Considering the softness of sintered Ag hydrogel, it may undergo extreme and repeated deformation when used on the mucosa. The resistance of the sintered Ag hydrogel remained around 1 Ω when stretched to 400%, which is capable of enduring the extreme deformation (Fig. 3G). In general, cyclic stretching can also cause detrimental effects on the electrical conductivity of stretchable composite conductors [47]. The sintering process and double network endow the sintered Ag hydrogel with an invariant resistance (*R*/*R*_0_ = 0.88) during 50% repetitive stretching (Fig. S11). The Young’s modulus of the sintered Ag hydrogel (around 91 kPa) is still much softer than that of the gastrointestinal tract (0.5 MPa to 1.2 MPa) [48] (Fig. 3H and Movie S1). After the sintering process, the Young’s modulus of the Ag hydrogel increased because the Cl^−^ was recognized as a kosmotropic ion in the context of the Hofmeister effect to enhance the salting-out phenomenon and aggregation of SA within the PAAm–SA matrix [49,50].

Meanwhile, considering the body fluid deployment environment on the mucosa, Ag hydrogels were soaked in different digestive juices (Fig. 3I). The resistance of the Ag hydrogel without sintering, soaked in AIF, increased from 1 Ω to 10^5^ Ω over time, whereas the sintered Ag hydrogel stably maintained a resistance below 1 Ω in AIF. The sintered Ag flakes formed a stable electron transfer pathway that can tolerate hydrogel swelling. Intriguingly, when the sintered Ag hydrogel and Ag hydrogel without sintering were immersed in SGF, both resistances remained around 1 Ω because Cl^−^ and H^+^ in SGF are effective components of the sintering solution. This result was also applicable to fluids that contain H^+^ and Cl^−^ (Fig. S12). The electrical stability of the hydrogel was evaluated using an ex vivo porcine stomach ablation model. Quantitative measurements showed that the electrical conductivity was largely preserved after IRE treatment, with a minor decrease from (1.77 ± 0.19) × 10^4^ S/m before ablation to (1.48 ± 0.26) × 10^4^ S/m after ablation, with no significant difference observed (*n* = 5, *p* > 0.05). These results indicate that the sintered Ag hydrogel retained its electrical functionality under high-voltage pulse exposure (Fig. S13). In addition, the biocompatibility of the sintered Ag hydrogel was evaluated in vitro and in vivo. In the cytotoxicity assessment, cells cultured in the sintered Ag hydrogel environment show high cell viability, exceeding 95% after 24 h of coincubation (Fig. 3J-i). Cells exposed to sintered Ag-hydrogel-soaked Dulbecco’s modified Eagle’s medium exhibited green fluorescence from viable cells comparable to that of the negative control group (Fig. 3J-ii and Fig. S14). The biocompatibility in the rat model following the standard procedure showed no evident tissue damage [51] (Fig. S15).

Although metallic Ag additives carry potential toxicity risks in body fluids due to Ag ion release, the relatively large micrometer-scale dimensions and reduced Ag content due to the sintering solution collectively mitigate potential cytotoxicity [52,53]. Ag ion release experiments showed that in SGF at 37°C, Ag ion release gradually increased over 60 min but remained at a very low concentration of only 30.86 μg/L. Even when the incubation time was extended to 24 h and 72 h, the Ag ion concentrations were only 31.25μg/L and 31.39 μg/L, respectively (Fig. S16). Besides, the total contact period between the sintered Ag hydrogel and gastric mucosa in a general ablation operation was less than 30 min, further avoiding the risk of potential toxicity of Ag. We placed the sintered Ag hydrogel in the stomachs of rats for 1 and 24 h to assess its accumulation in organs such as the heart, liver, spleen, lungs, and kidneys. Notably, Ag concentrations in all organs were very low (Fig. S17). Ag concentrations in the kidneys of the control group were even higher than those in the experimental group. The highest concentration was 15.35 μg/kg, which is lower than the physiological silver concentration reported for human liver and kidney tissues (50 μg/kg) [54]. Hematoxylin and eosin (H&E) sections of these organs also revealed no signs of damage (Fig. S18). Therefore, the sintered Ag hydrogel is safe and feasible for optimizing the bioelectronic tissue interface in mucosal IRE.

The adhesion properties of the sintered Ag hydrogel were evaluated by lap shear testing. The shear stress between the sintered Ag hydrogel and the stomach was 17.83 kPa, and that between the sintered Ag hydrogel and the metal electrode was 21.7 kPa (Fig. S19). Although this adhesion strength is sufficient to reach the stomach, there is a risk of electrode detachment during the entire ablation process in the complex gastric environment [55]. Therefore, magnetic anchoring was used in in vivo experiments to enhance the adhesion between the sintered Ag hydrogel and the mucosa during ablation.

### Evaluation of enhanced bioelectronic tissue interface by the sintered Ag hydrogel

We designed a planar flexible electrode that is suitable for mucosal IRE, with the hydrogel interface located on the surface of the metal electrode (Fig. 4A). Based on our previous work, this type of electrode structure can achieve an ablation zone with a controllable range and depth by adjusting the circuit morphology and pulse settings [10]. Fig. 4B illustrates the charge transfer mechanisms under different electrode-tissue interface conditions. Charges are transferred through electron flow in the metal electrode, whereas they are transferred via ion flow in tissue. The sintered Ag hydrogel interface in this study can not only establish an electron transfer network with the metal electrode through Ag flakes but also construct a charge exchange network with tissue using ions in the matrix, ultimately enabling efficient electric field delivery between bioelectronics and tissue. Therefore, the sintered Ag hydrogel interface demonstrated enhanced bioelectronic tissue integration by mimicking tissue compliance and efficiently delivering the electric field. These adaptabilities are critical for establishing a stable mechanical and electrical coupling of the electrode-tissue interface during IRE.

**Fig. 4.**
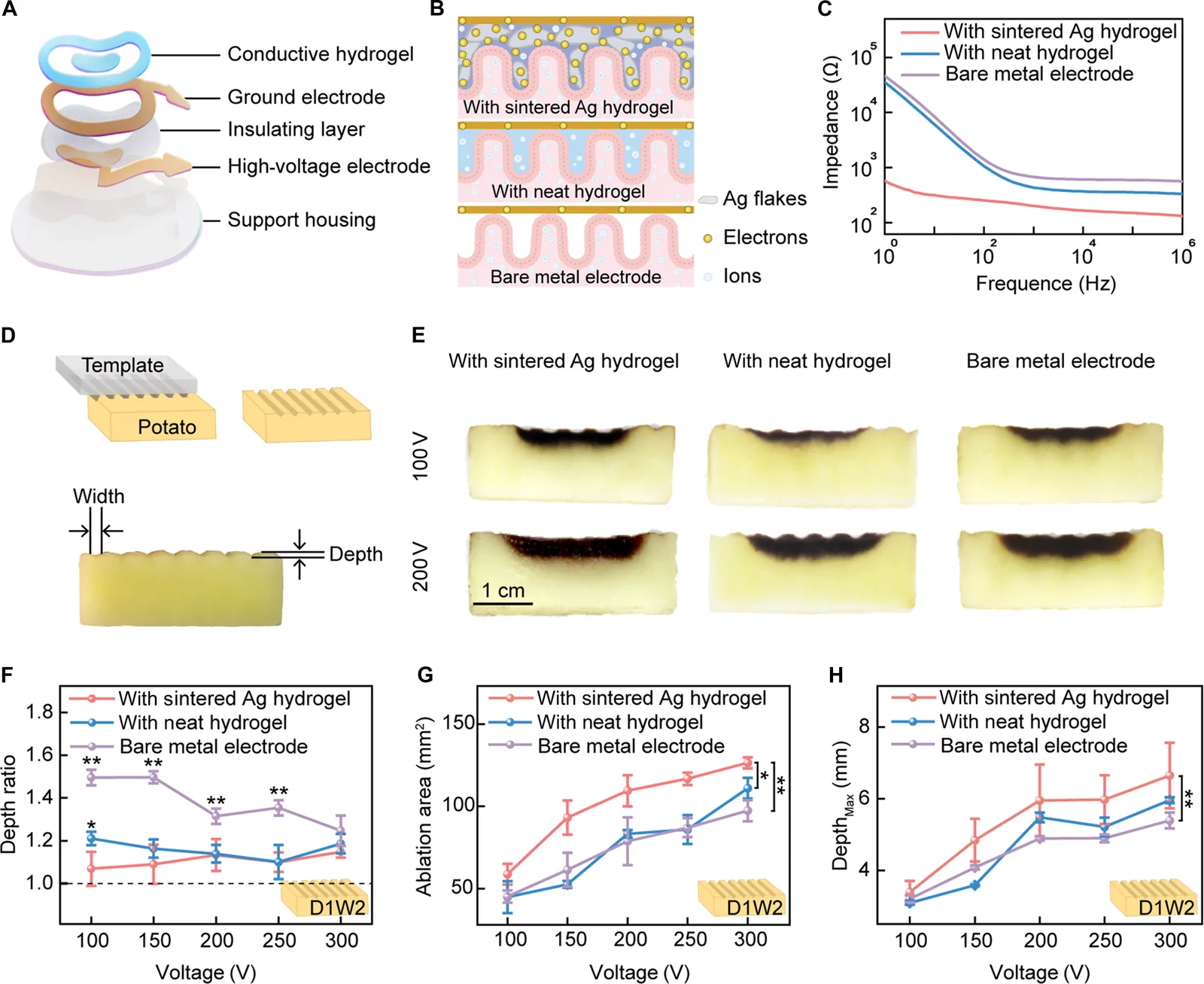
Evaluation of enhanced bioelectronic and tissue interface by the sintered Ag hydrogel. (A) Schematic diagram of flexible electrode structure with a hydrogel interface. (B) Schematic of the charge transfer mechanism of the electrode with a sintered Ag hydrogel, the electrode with neat hydrogel, and the bare metal electrode in contact with the gastric mucosa (created in BioRender). (C) The contact impedance of the electrode and mucosa under different interface conditions at frequencies ranging from 1 to 10^6^ Hz. (D) Schematic diagram of the modeling method and the image of the potato model. (E) Cross-sectional images of the ablated potato cubes. (F) The ablation depth ratio, (G) the ablation area, and (H) the maximum ablation depth under different interface conditions in 1-mm-depth, 2-mm-width groove models, respectively. *p < 0.05; **p < 0.01.

We further validated the optimization effect of conductive hydrogels on the electrode-tissue interface using in vitro tissue models. Electrochemical impedance spectroscopy was used to investigate the function of the hydrogel and the significance of high conductivity in reducing the charge transfer barrier between the electrode and mucosa. The electrode with the sintered Ag hydrogel interface achieved remarkably low contact impedance with a porcine gastric tissue sample across frequencies, particularly below 100 Hz (Fig. 4C). At 10 Hz, the impedance remained near 10^2^ Ω, outperforming bare metal electrodes (>10^4^ Ω) and electrodes with neat hydrogel interfaces (~10^4^ Ω). The neat hydrogel can reduce interface impedance due to the benefit of eliminating dead spaces, but its intrinsically low ionic conductivity (<0.1 S/m) still poses a barrier to charge transfer. Overall, the sintered Ag hydrogel, with both softness and electron-dominated conductivity (>10^2^ S/m), is an ideal material for reducing the charge transfer barrier between the electrode and the mucosa during IRE.

The uniformity of the ablation was evaluated using in vitro potato models (Fig. S20). The potato is one of the most commonly used phantom models, as it clearly shows the ablation area after IRE [56,57]. This is mainly because the oxidation of intracellular polyphenol efflux from the destroyed cells can lead to melanin buildup and the formation of blackened spots [58]. Although the Young’s modulus of potato is much higher than that of the stomach, its surface can be cut and shaped into a rough morphology similar to the mucosa. In addition, potato samples can be mass-produced to enable standardized quantitative experiments. Therefore, homogeneous potato samples were cut into 30 mm × 20 mm × 10 mm uniform cubes, and grooves with defined dimensions were precisely cut using 3D-printed blades as a phantom model in this study (Fig. 4D).

In our work, the evaluation of ablation uniformity and efficiency primarily involves 3 parameters: the ablation depth ratio on rough surfaces, which indicates the ablation uniformity; and the ablation area and the maximum ablation depth, both of which serve as indicators of ablation efficiency. Fig. 4E shows the distribution of electrical injury at the central cross-section of potato models under different interface conditions after IRE (from left to right: electrode with sintered Ag hydrogel, electrode with neat hydrogel, and bare metal electrode). The images demonstrated that the sintered Ag hydrogel interface resulted in a more uniform ablation, a well-defined boundary, and the largest ablation area. The ablation depth ratio was defined as the most protrusive ablation depth in the central area over the ablation depth of the neighboring concave region (Fig. S21). The ablation depth ratio reflected the discrepancy in ablation depth across the model’s adjacent regions, and the value of 1 means completely uniform ablation. Gastric mucosa lesions typically exhibit irregular morphologies, resulting in variable infiltration depths within the gastric wall. A higher ablation depth ratio indicates poor ablation uniformity on irregular surfaces, potentially resulting in irregular ablation boundaries and residual cells that are not fully ablated. Conversely, an ablation depth ratio approaching unity indicates improved ablation uniformity on such surfaces, with ablation boundaries approaching a smooth and straight line.

Fig. 4F shows that, under all parameter-setting conditions, the sintered Ag hydrogel interface group has the lowest ablation depth ratio, while the bare electrode group has the highest. Interestingly, although the ablation depth ratio of the neat hydrogel interface group is slightly higher than that for the sintered Ag hydrogel group, it still demonstrates a significant improvement over the bare metal electrode group. This firmly confirmed the importance of the electrode-mucosa interface for achieving a homogeneous distribution of the electric field and electrical injury. The experimental results of the tissue phantom are consistent with those of the simulation analysis (compare Fig. 4E with Fig. 2C-iii). Furthermore, the variation in ablation depth ratios among different interface conditions exhibits a declining trend as pulse voltage increases, indicating that pulse voltage positively affects ablation uniformity. Overall, it has been confirmed that the sintered Ag hydrogel improved ablation uniformity by 40% to 86%, reduced the variation in ablation depth, and resulted in the ablation boundary being closer to a continuous, smooth straight line.

Fig. 4G shows that the sintered Ag hydrogel interface group has the largest ablation area. Compared to the bare metal electrode, the sintered Ag hydrogel interface significantly increased the ablation area by 30% to 52%. Importantly, the ablation area of the bare electrode group is roughly the same as that of the neat hydrogel group, both of which are much lower than that of the sintered Ag hydrogel group. These results indicated that although a highly conductive sintered Ag hydrogel or an ionic conductive neat hydrogel reduced the ablation depth ratio by eliminating the dead space at the interface, the lower conductivity of the neat hydrogel weakened the electric field intensity, resulting in a significantly reduced ablation area compared to that of the sintered Ag hydrogel interface.

The maximum ablation depth serves as a critical parameter for evaluating the efficiency of ablation on the gastric wall. An increased ablation depth range indicates enhanced controllability of mucosal ablation, extending the potential application to various types of lesions. As depicted in Fig. 4H, the sintered Ag hydrogel interface group exhibits the most profound ablation depth among the groups tested. In addition, the advantage of the sintered Ag hydrogel interface was more pronounced when the depth and width of the grooves on potato models were increased. When the groove’s dimension doubles, the ablation depth ratio under the bare electrode condition increases nearly twice (1.45 to 2.80), while that under the Ag hydrogel condition remains at a low level of 1.04, indicating its versatility in achieving uniform ablation on rough tissue surfaces with diverse morphologies (Figs. S22 and S23).

The potato model allows for the rapid and systematic quantification of ablation trends across different interfaces, providing a reference for in vivo experiments. The results presented above indicate that the ablation area and depth achievable with a bare electrode at high voltage can be achieved at lower voltage when utilizing the sintered Ag hydrogel interface, which is anticipated to enhance the safety profile of IRE. Furthermore, increased ablation depth enhances surgeons’ confidence in mucosal IRE for large-volume lesions, as it mitigates concerns regarding complication risks under relatively lower high-voltage conditions. Overall, it is essential to establish an interface between the electrode and the mucosa that combines both softness and high electrical conductivity to ensure uniform and complete mucosal IRE.

### Preclinical validation for gastric mucosa IRE via endoscopy by the sintered Ag hydrogel interface

Preclinical validation of gastric mucosa IRE via endoscopy in a porcine model was conducted to further demonstrate the enhanced ablation uniformity and efficacy enabled by the sintered Ag hydrogel interface. As shown in Fig. 5A, IRE was conducted on the body of a porcine stomach, where gastric pits with a rough surface were distributed. The IRE was conducted by a magnetic-anchoring-assisted EACS, which included a tip-mounted metal electrode (Fig. 5B-i). The metal electrode with the sintered Ag hydrogel interface was integrated onto the flexible catheter, and the ablation operation was performed by standard endoscopic surgery (Fig. 5B-ii and iii and Fig. S24). The electrode was guided into the stomach along with the endoscope by grasping the ring on the back of the TPU-supporting substrate with grasping forceps (Fig. S25 and Movie S2). When the electrode approached the targeted region, the magnetic anchoring technique was used to ensure tight contact between the sintered Ag hydrogel interface and mucosa during endoscopic surgery (Fig. S26A). An internal magnet was embedded within the TPU-supporting substrate to bring in compression force between the EACS and the mucosa through the magnetic force generated by an external magnetic device (Fig. S26B). The integrated design can ensure both the precise location and stable anchoring of the EACS in the stomach.

**Fig. 5.**
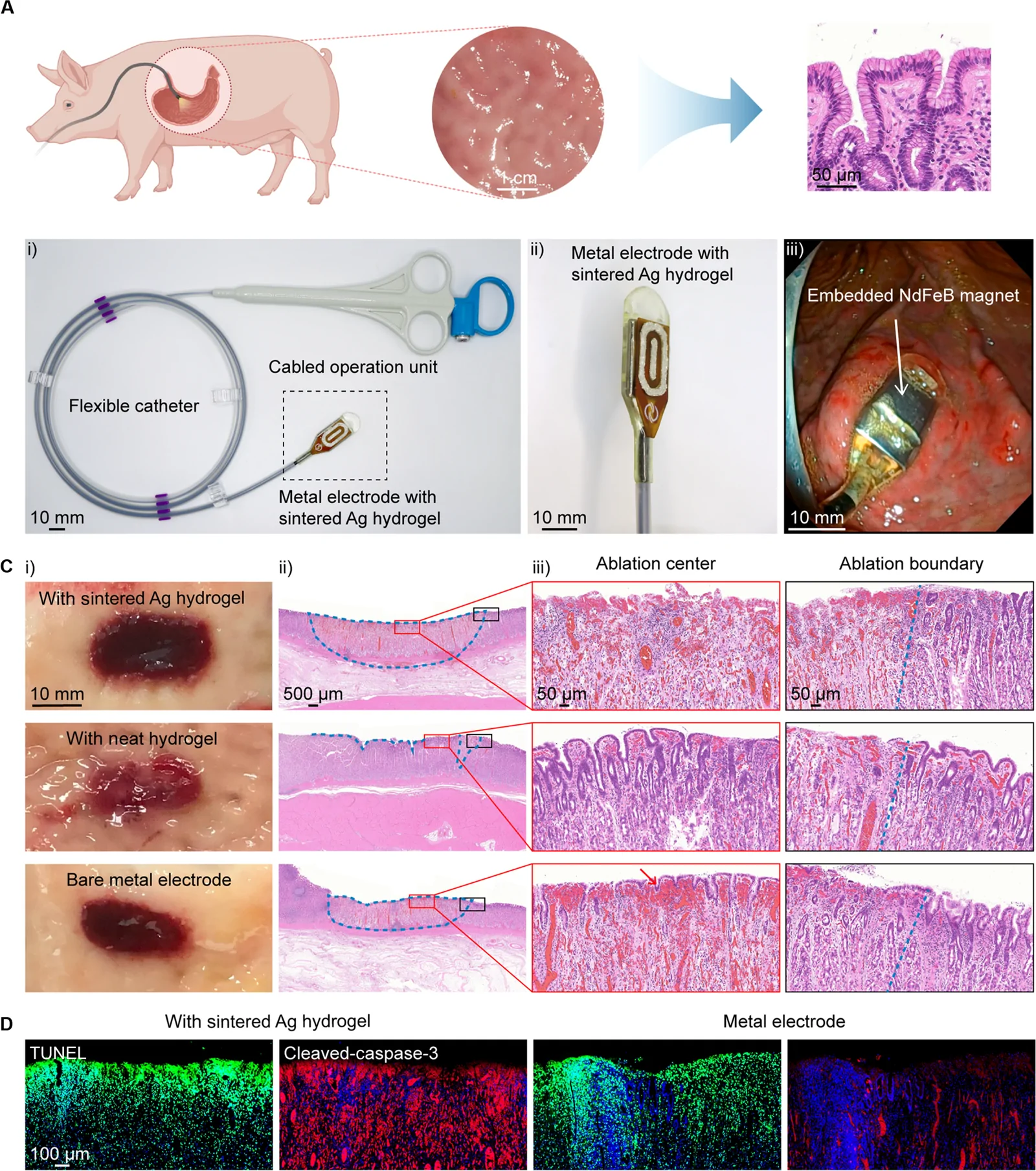
Preclinical validation for endoscopic gastric mucosal ablation by endoscopic ablation catheter system (EACS) with a sintered Ag hydrogel interface. (A) Schematic diagram of the in vivo irreversible electroporation (IRE) setup on a porcine stomach (left), highlighting the uneven gastric-pit-distributed surface (center), and the histological image of normal gastric mucosa (right) (the illustration was created in BioRender). (B) (i) The images of magnetic-anchoring-assisted EACS. (ii) The metal electrode with a sintered Ag hydrogel interface. (iii) Endoscopic view of the metal electrode with a sintered Ag hydrogel interface in the porcine stomach. (C) (i) The top-view appearance of the mucosal surface after IRE under different interface conditions (from the top to the bottom: electrode with a sintered hydrogel interface, with a neat hydrogel interface, and bare electrode). (ii) The hematoxylin and eosin (H&E) staining images. Scale bar, 500 μm. (iii) The H&E staining images of the central and boundary parts of the ablated mucosa. Scale bars, 50 μm. (D) Representative TUNEL images (scale bar, 100 μm) and cleaved-caspase-3 images (scale bar, 100 μm) of the mucosa ablated by the metal electrodes with sintered Ag hydrogel interface and bare metal electrodes (TUNEL, green; cleaved-caspase-3, red; 4′,6-diamidino-2-phenylindole, blue).

Fig. 5C shows the results of gross examination and histopathological analysis of mucosal IRE with or without a conductive and conformal interface. As shown in Fig. 5C-i, under the same pulse parameters and magnetic-anchoring-assisted conditions, the ablation zone displays a more regular and distinct boundary, a larger ablated area, and more severe electrical injury (dark red) with the sintered Ag hydrogel interface. The controllable ablation zone facilitates precise ablation adjustment based on lesion size during gastric mucosal ablation procedures. In contrast, the bare metal electrode had only partial contact with the gastric mucosa, resulting in a limited ablation area and an irregular ablation boundary. In addition, the neat hydrogel, which was deficient in conductivity, weakened the delivery of the electric field.

The corresponding H&E staining images revealed the histopathological characteristics of postablation tissues along the long-axis section of the ablation zone (Fig. 5C-ii). Under the same pulse voltage condition, the metal electrode with a sintered Ag hydrogel interface group exhibits the largest area, the most profound ablation depth, and the most distinct boundary. Moreover, only under the Ag hydrogel interface condition can electrical injury reach the muscularis mucosae (~2.50 mm in depth), enabling complete ablation of mucosal lesions, which is of great significance for ensuring the ablation efficacy. The sintered Ag hydrogel achieved the deepest ablation depth, and the difference in ablation depth compared to the other 2 groups was more pronounced than the results from simulations and in vitro experiments (Fig. S27). The ablation zones of the neat hydrogel electrode group are too small to form a continuous, large-scale ablation zone (the region outlined by the blue dashed line), which may suggest that the conductivity of the bioelectronic tissue interface is critical during actual clinical applications.

Furthermore, we compared histopathological differences between the interior of the ablation zone and the ablation boundary (Fig. 5C-iii, left: ablation center and right: ablation boundary). In the Ag hydrogel interface group (Fig. 5C-iii, top), within the central ablation region, mucosal epithelial cells display a disorganized arrangement, with the disappearance of original glandular structures. Extensive infiltration of red blood cells is seen throughout the mucosa. Staining of the mucosal epithelium is diminished, nuclear details are absent, and cellular integrity is compromised, with no identifiable intact residual mucosal epithelial cells. These findings unequivocally indicate complete ablation of the mucosal layer. In the boundary region, a clear demarcation exists between the damaged area and the adjacent undamaged tissue, with the damaged area devoid of viable cells. Conversely, cells in the undamaged region retain intact morphological structures and demonstrate normal staining patterns, showing no evidence of injury from the electric field.

In the bare metal group (Fig. 5C-iii, bottom), the central ablation region shows a disorganized arrangement of mucosal cells, with focal clusters of blood cells. Despite this, intact mucosal cell structures remain visible on the surface, indicating the persistence of residual viable cells within the central region. In the boundary region, the distinction between the severely damaged and undamaged areas is not clearly defined. Although mucosal surface cells are compromised, scattered viable cells can still be detected within the deeper layers.

Within the neat hydrogel interface group (Fig. 5C-iii, middle), intact mucosal glandular structures and mucosal cells exhibiting preserved morphology and clear staining are observable in the central region, concomitant with a widespread distribution of viable cells and localized cellular damage. This observation suggests incomplete ablation, which may compromise the uniformity and efficacy of the ablation-induced tissue injury. In the boundary region, the ablation margin cannot be distinctly delineated because of impaired electric field delivery. In addition, focal accumulations of blood cells and residual mucosal cells with preserved structural integrity are present on both sides of the presumed boundary.

The fluorescence staining of terminal deoxynucleotidyl transferase–mediated deoxyuridine triphosphate nick-end labeling (TUNEL) and cleaved-caspase-3 showed that IRE induced cell apoptosis through activating the caspase-dependent pathway (Fig. 5D and Fig. S28). The sintered Ag hydrogel interface group exhibited the highest number of TUNEL-positive cells, with extensive, contiguous areas of accumulation. In contrast, the bare metal electrode group showed a highly inhomogeneous distribution of TUNEL-positive cells, with residual cells in the ablation zone, indicating incomplete ablation. There was no evident acute histological abnormality in the examined submucosa and muscularis propria directly opposite the ablation site at 1 h postablation (Fig. S29).

In addition, we evaluated changes in mucosal bioimpedance spectroscopy before and after ablation. The decrease in impedance was observed across all ablated gastric wall regions, particularly at low frequencies, consistent with increased tissue electrical permeability following electroporation-induced membrane permeabilization (Fig. S30) [59]. Following in vivo ablation, the retrieved sintered Ag hydrogel maintained its macroscopic appearance with preserved shape and overall integrity after IRE (Fig. S31). SEM characterization further revealed preserved hydrogel microstructure and the distribution of embedded Ag flakes after IRE exposure (Fig. S32). Together, these findings support the electrical and structural stability of the sintered Ag hydrogel during IRE. The preserved macroscopic morphology and microstructural characteristics of the hydrogel after IRE suggest that the overall dual-network architecture, including the calcium-ion-cross-linked SA component, tolerated high-voltage pulse exposure without apparent structural disruption. Furthermore, we used a rat model to compare the ablation area under different interface conditions, aiming to compensate for the deficiency caused by measurement errors due to the elastic deformation of gastric wall samples before and after sampling. Consistently, we found that the ablation area under the sintered Ag hydrogel interface condition was significantly larger than that in other groups (Fig. S33).

We conducted a systematic comparison of sintered Ag hydrogel electrodes with existing IRE electrodes across 5 dimensions (Table S2), including balloon electrodes, needle electrodes, poly(3,4-ethylenedioxythiophene)-coated electrodes, and conventional planar metal electrodes. The sintered Ag hydrogel electrode exhibits enhanced mechanical compliance and improved conformability, which may facilitate adaptation to complex tissue geometries. Although the electrical conductivity of sintered Ag hydrogel is lower than that of other electrodes, it is sufficient for ablation applications.

## Discussion

In summary, we achieved uniform and complete gastric mucosal IRE by introducing a sintered Ag hydrogel as the bioelectronic tissue interface between the electrode and the mucosa. Simulation analysis was used to theoretically demonstrate the positive role of conductive hydrogels in optimizing the bioelectronic tissue interface during IRE. The invented room-temperature sintering process in Ag-flake-filled hydrogel enhances the electron transfer pathways, ensuring low Young’s modulus (~91 kPa) and high electrical conductivity (>10^4^ S/m) even under conditions of swelling in digestive juice and mechanical deformation. Subsequently, in vitro experiments utilizing potato phantom models demonstrated that the Ag hydrogel interface significantly enhances ablation uniformity by 40% to 86%, extends the ablation area by 30% to 52%, and increases the maximum ablation depth up to 5.39 mm. The efficacy of the sintered Ag hydrogel interface was further validated in a preclinical porcine model by integrating it with a magnetic-anchoring device, called the EACS. The sintered Ag hydrogel, characterized by its appropriate softness and high electrical conductivity, facilitates conformal contact with the electrode and the mucosa, enabling efficient electric field delivery without attenuation. Ultimately, this results in uniform and complete mucosal IRE, with an ablation depth reaching up to 2.50 mm (500 V) and complete coverage of the muscularis mucosae, making it a promising endoscopic treatment option for gastrointestinal lesions.

IRE is a promising electrotherapy modality that typically uses needle-shaped ablation electrodes for treating solid tumors and catheter-based basket-shaped electrodes for atrial fibrillation. However, mucosal ablation via endoscopy, as a unique application scenario, has historically lacked suitable solutions. The EACS, incorporating a room-temperature sintered Ag hydrogel interface, effectively mitigates the mechanical mismatch between metal electrodes and the soft, uneven, and moist mucosa, while also enhancing electric field delivery efficiency within the gastric mucosa. Notably, in this study, the interface optimization provided by the sintered Ag hydrogel enables ablation depths of up to 2.50 mm in the porcine model. This indicates that localized ablation reaching the muscularis mucosae can be performed effectively without compromising the structural integrity of the gastric wall, which also provides a theoretical possibility for applying IRE to other scenarios, such as the biliary–pancreatic duct and airway.

Despite these promising preclinical findings, several limitations warrant consideration. First, the optimal electrical conductivity required for a hydrogel interface may vary across different mucosal tissues and remains to be established. Second, the adhesive interactions among the interface material, mucosa, and electrode require further optimization. Third, further development is required before clinical translation of the present platform. The current prototype uses an endoscopically guided over-the-scope configuration and combines a conductive hydrogel interface, a planar electrode, magnetic anchoring, and dedicated positioning procedures. This multicomponent workflow may increase procedural complexity and require further optimization to improve workflow efficiency and adaptability in endoscopic settings. In the present acute porcine study, magnetic anchoring was used as a prototype-specific stabilization strategy to help maintain electrode–mucosa contact and a consistent ablation interface. However, it introduces an additional procedural step, and its design requires further optimization to simplify deployment and improve adaptability across different anatomical locations. Future translation will require greater integration and simplification of the hydrogel interface, electrode, fixation, and delivery components, including improved compatibility with conventional endoscopic platforms. Finally, the enhancement of IRE efficacy by the sintered Ag hydrogel interface should be further evaluated in more clinically representative settings, including lesions with varied sizes and morphologies, and ultimately in clinical studies.

Overall, our work addressed one of the key challenges in completely treating the mucosal lesion while preserving the structural and functional integrity of the stomach by utilizing IRE with a sintered Ag hydrogel as the bioelectronic tissue interface. We performed simulation analysis for theoretical verification, designed and developed a room-temperature sintering process to produce a soft, highly conductive hydrogel, and conducted both in vitro and in vivo experiments after integrating the hydrogel interface with the EACS device, highlighting the potential of IRE for future treatment of mucosa-related diseases.

## Data Availability

All data are available in the main text or the Supplementary Materials.

## Supplementary Materials

Supplementary 1 Figs. S1 to S33 Tables S1 and S2 Movies S1 and S2 References [60–66]
